# Identifying the most important workplace factors in predicting nurse mental health using machine learning techniques

**DOI:** 10.1186/s12912-021-00742-9

**Published:** 2021-11-01

**Authors:** Farinaz Havaei, Xuejun Ryan Ji, Maura MacPhee, Heather Straight

**Affiliations:** 1 University of British Columbia (UBC), School of Nursing, 201-2211 Wesbrook Mall, Vancouver, BC V6T 2B5 Canada; 2 UBC Department of Educational and Counselling Psychology, and Special Education, 2125 Main Mall, Vancouver, BC V6T 1Z4 Canada; 3 British Columbia Nurses Union, 4060 Regent Street, Burnaby, BC V5C 6P5 Canada

**Keywords:** Mental health, Work environment factors, Nursing, National standard of psychological health and safety, Machine learning

## Abstract

**Objectives:**

Nurses are at a high risk of developing mental health problems due to exposure to work environment risk factors. Previous research in this area has only examined a few factors within nurses’ work environments, and those factors were not conceptualized with the goal of improving workplace mental health. The purpose of this study is to identify the most important work environment predictors of nurse mental health using a comprehensive and theoretically grounded measure based on the National Standard of Psychological Health and Safety in the Workplace.

**Methods:**

This is an exploratory cross-sectional survey study of nurses in British Columbia, Canada. For this study, responses from a convenience sample of 4029 actively working direct care nurses were analyzed using random forest regression methods. Key predictors include 13 work environment factors. Study outcomes include depression, anxiety, post-traumatic stress disorder (PTSD), burnout and life satisfaction.

**Results:**

Overall, healthier reports of work environment conditions were associated with better nurse mental health. More specifically *balance*, *psychological protection* and *workload management* were the most important predictors of depression, anxiety, PTSD and emotional exhaustion. While *engagement*, *workload management*, *psychological protection* and *balance* were the most important predictors of depersonalization, *engagement* was the most important predictor of personal accomplishment. *Balance*, *psychological protection* and *engagement* were the most important predictors of life satisfaction.

**Conclusions:**

Routine assessment with standardized tools of nurses’ work environment conditions and mental health is an important, evidence-based organizational intervention. This study suggests nurses’ mental health is particularly influenced by worklife balance, psychological protection and workload management.

## Background

Nurses and other healthcare providers are at a high risk of developing mental health problems due to significant exposure to workplace risk factors such as human suffering and death, inadequate staffing, heavy workloads and workplace violence [1, 2]. Poor mental health in healthcare workers has been linked to sub-optimal performance [3], negative patient safety outcomes [4], staff absenteeism [3] and turnover [5]. International research has found that workplace conditions such as workload management, staffing and resource adequacy are among the most important determinants of nurse mental health; however, this body of evidence is limited in that it has only examined a few factors within nurses’ work environments and those factors were not conceptualized with the goal of improving workplace mental health [6–8]. The purpose of this study is to identify the most important workplace conditions predicting the mental health of nurses, the largest health human resource worldwide, using a theoretically sound and comprehensive measure of work environment conditions that was developed with the intent of optimizing workplace mental health based on the National Standard for Workplace Psychological Health and Safety” (i.e., the Standard) [9, 10]. Given the expected shortage of the nursing workforce in Canada and internationally [11, 12], this research is both timely and important as it helps identify and address aspects of the workplace associated with nurses’ mental health—an important dimension of nurse job satisfaction and retention [13].

### Mental health

The World Health Organization defines mental health as “a state of well-being in which an individual realizes his or her own abilities, can cope with the normal stresses of life, can work productively and is able to make a contribution to his or her community” [14]. Thus, mental health can be operationalized through the presence or absence of a general positive state or mental health disorders such as depression, anxiety, Post Traumatic Stress Disorder (PTSD) and burnout. The prevalence of these mental health problems has been found to range between 10% (PTSD) and 41% (anxiety) among different groups of nurses worldwide [15]. In Canada, a national pre-pandemic survey showed that 20% of 7358 participating nurses met the criteria for PTSD and general anxiety disorder; and one third met the criteria for major depressive disorder [1]. Using a subsample of this data, Stelnicki and Carlton found jurisdictional differences in nurse mental health; nurses in Eastern Canada (i.e. Ontario and Quebec) were more likely to report mental health problems compared to their peers in Western jurisdictions (i.e. British Columbia, Alberta, Saskatchewan and Manitoba) [16]. When analyzed separately, British Columbia (BC) nurses were found to have more concerning mental health than their peers nationally; using pre-pandemic data, a 2021 study showed the prevalence of mental health problems as 1.5 to 3 times higher among BC nurses compared to their peers nationally [15].

### Work environment influences

Work environment conditions play a major role in influencing nurses’ mental health [6, 17–21]. Internationally, the nursing work environment literature can be classified into three distinct bodies of evidence: (a) structural empowerment [22–24], (b) magnet hospitals [6, 25], and (c) the areas of worklife [7, 8, 26]. Building on Kanter’s theory of power within organizations, Laschinger et al. developed the notion of work environment structural empowerment [27]. Measured by the Conditions of Work Environment Questionnaire II (CWEQ-II), structural empowerment refers to nurses’ perceptions of access to six empowering work environment structures including information, resources, opportunities, supports and formal and informal channels of power [28]. Another body of literature focuses on the concept of magnet hospital, which originated in the early 1980s during a severe nursing shortage in the United States. Some hospitals, known as “magnet hospitals,” had certain characteristics associated with better nurse recruitment and retention than non-magnet hospitals [6, 25]. Building on this initial research, Lake identified five attributes of magnet hospitals: leadership, collegial nurse-physician relations, opportunities for nurse participation, adequacy of staffing and resources as well as a nursing (rather than a biomedical) model of care. These attributes can be measured by the Practice Environment Scale from the Nursing Work Index (PES-NWI) [6, 25]. The third body of work environment evidence, the Areas of Worklife Model, originated from the work of Maslach and Leiter on nursing burnout [7, 8] According to this body of evidence, a mismatch between individual nurses and six aspects of their work environment is the main antecedent of burnout. Operationalized by the Areas of Worklife Survey (AWS), these work environment aspects include workload, control, reward, community, fairness and values [7, 29]. Despite their impact on nurse mental health and wellbeing, these conceptualizations of work environment conditions were not developed with the goal of protecting nurses’ mental health in the workplace.

The Mental Health Commission of Canada developed the Standard which is comprised of a set of guidelines, tools and resources aimed at promoting employee health and preventing mental health injury in the workplace [9, 10]. While the Standard is not specific to nurses or the healthcare sector, it has been developed with the goal of improving worker mental health [9, 10].

In recognition of the rising mental injury among nurses and other healthcare providers, in 2016, the British Columbia Nurses Union (BCNU) became the first and only union in Canada to bargain for implementation of The Standard within the province’s health authorities [30]. As a result of this bargaining effort, BC health authorities are mandated to protect the mental health of their nursing workers and other healthcare providers in the workplace via the implementation of the Standard.

The implementation of the Standard begins with a comprehensive, baseline assessment of work environment conditions most important to employee mental health. Founded upon empirical and theoretical evidence, these conditions were systematically identified using a grounded theory approach which involved a comprehensive literature review and consultation with subject matter experts with the intent of optimizing mental health in the workplace [9, 31]. A 13-factor measure, the Guarding Minds at Work (GMW) Survey, was developed to measure workplace conditions important to employee mental health. The GMW was recently validated among 3077 direct care nurses working in acute care settings in BC [30]. This study found that pre-pandemic over half of the nurse respondents were concerned about nine of the 13 workplace factors assessed using the GMW survey [30]. The current study examines one key research question: which of the Standard’s 13 workplace conditions most strongly predict nurses’ mental health?

## Methods

This study is a partnership between nurse researchers and the BCNU representing nearly 48,000 nurses in the province using a cross-sectional correlational survey design. The BCNU sent an email invite with the survey link to its nurse members asking them to complete the study survey. Participants were informed of the voluntary nature of their participation; the confidentiality of their responses and that survey submission would indicate informed consent. To increase response rate, a series of strategies were used including a two-month data collection period, weekly reminders, survey advertisement on multiple platforms and a raffle draw for two Apple Watches. In total, 5512 surveys were returned resulting in an estimated 12% response rate. For this study, only actively working direct care nurses were included yielding a final sample size of 4029 participants. Ethics approval was obtained from the University Behavioral Research Ethics Board (approval number: H18–02724).

### Measures

#### Outcomes

Mental health was operationalized as depression, anxiety, PTSD, burnout (three subscales) and life satisfaction scores. Depression was measured with the Patient Health Questionnaire (PHQ-9) comprised of nine items rated on a four-point scale ranging from 0 (not at all) to 3 (nearly every day) [32]. Anxiety was measured with the General Anxiety Disorder Scale (GAD-7) consisting of seven items rated on a four-point scale ranging from 0 (not at all) to 3 (nearly every day) [33]. PTSD was measured with the Post-Traumatic Stress Syndrome 14-Questions Inventory (PTSS-14) comprised of 14 items rated on a seven-point scale ranging from 1 (never) to 7 (always) [34]. Burnout was measured by the 22-item Maslach Burnout Inventory-Human Services Survey (MBI-HSS) comprised of three subscales: Emotional Exhaustion (EE, nine items), Depersonalization (DP, five items) and Personal Accomplishment (PA, eight items) [35]. Additionally, a single life satisfaction indicator, adopted from Statistics Canada, asked participants to rate the extent to which they were satisfied or dissatisfied with their life as a whole with response options ranging from very dissatisfied (0) to very satisfied [10, 36]. Guided by previous research, the first four mental health outcomes were converted into composite factor scores using Confirmatory Factor Analysis (CFA) [37, 38]. This technique was not applied to the measure of life satisfaction since it is a single variable index.

#### Predictors

The conditions of nurses’ work environments were measured using the GMW survey consisting of 65 items and 13 factors (Table 1). Each factor consists of five statements about a specific workplace condition, and participants are asked to indicate their level of agreement or disagreement with each statement on a four-point scale ranging from strongly disagree [1] to strongly agree [4]. The internal structure of the measure was previously evaluated among BC nurses and yielded a 13-factor structure [30]. In this study, because we are interested in the predictive power of each work environment factor, composite factor scores were obtained using CFA [37, 38] with higher factor scores indicating healthier workplace conditions.

**Table 1:** Descriptive statistics and reliability coefficients for the main study variables

Measures	n	M	SD	Min	Max	McDonald’ ω	95% CI	
							LL	UL
**Workplace Predictors**								
Psychological Support	4029	0.00	0.45	−0.95	0.93	0.82	0.81	0.83
Organizational Culture	4029	0.00	0.52	−1.14	1.22	0.80	0.79	0.81
Leadership Expectations	4029	0.00	0.26	−0.53	0.56	0.82	0.81	0.83
Civility & Respect	4029	0.00	0.57	−1.33	1.19	0.82	0.81	0.83
Psychological Job Fit	4029	0.00	0.59	−1.56	1.28	0.74	0.72	0.75
Growth & Development	4029	0.00	0.53	−1.20	1.08	0.74	0.73	0.75
Recognition & Reward	4029	0.00	0.45	−0.79	1.03	0.82	0.81	0.83
Involvement & Influence	4029	0.00	0.52	−1.31	1.03	0.81	0.80	0.82
Workload Management	4029	0.00	0.69	−1.36	1.67	0.81	0.80	0.82
Engagement	4029	0.00	0.43	−2.04	0.52	0.80	0.79	0.81
Balance	4029	0.00	0.61	−1.17	1.37	0.81	0.80	0.82
Psychological Protection	4029	0.00	0.57	−0.95	1.26	0.86	0.86	0.87
Physical Safety	4029	0.00	0.72	−1.48	1.36	0.89	0.88	0.89
**Mental Health Outcomes**								
Depression	4029	0.00	0.65	−0.84	2.12	0.91	0.90	0.91
Anxiety	4029	0.00	0.77	−0.99	1.86	0.93	0.93	0.93
PTSD	4029	0.00	0.90	−1.51	2.66	0.93	0.93	0.93
Emotional Exhaustion	4029	0.00	1.43	−3.41	2.42	0.92	0.92	0.93
Depersonalization	4029	0.00	0.87	−1.18	2.19	0.82	0.81	0.82
Personal Accomplishment	4029	0.00	0.47	−2.02	0.78	0.78	0.77	0.79
Life Satisfaction	3737	6.74	1.86	0	10	–	–	–

#### Controls

A set of demographic variables such as age, gender (female, male), years of nursing experience, healthcare sector (acute, care, community care, long-term care) and geographical region (urban, suburban, rural) were included as control variables in this study. Healthcare sector and geographic region were dummy-coded as follows: (Healthcare sector, sec1: acute care = 1, long-term care = 0; sec2: community care = 1, long-term care =0; Geographical region, reg1: urban = 1, rural = 0; suburban =1, rural = 0).

### Data analysis

The internal consistencies for all the composite predictors and outcomes were evaluated using a coefficient ω > .8 as an indication of good internal consistency and a coefficient ω between .7 and .8 as an indication of acceptable internal consistency [39, 40]. The internal structure of the multi-item mental health outcomes was evaluated using a CFA approach with the following indices used to evaluate model fit: Comparative Fit Index (CFI ≥ 0.90), Tucker Lewis Index (TLI ≥ 0.95) and Standardized Root Mean Squared Residual (SRMR < 0.08) [41]. The internal structure of the GMW scale was recently evaluated using data from BC nurses in different study and therefore not included in the current study [30].

Data were further analyzed using random forest (RF) analysis, a supervised machine learning algorithm [42], that nonlinearly regressed each of the seven mental health outcomes on the 13 GMW factors after taking into account the impact of control variables including age, years of experience, healthcare sector and geographical region. As is the case with other RF studies [43], a 10-fold cross-validation was applied, and for each fold, a 70 and 30% dataset was respectively used as a training and testing set. The training and testing sets were then used to evaluate the model performance through the Root Mean Square Error (RMSE). A higher RMSE for the testing set compared to the training set would indicate the lack of overfit. The “importance score” of the predictors was gauged by the average level of the decline in model accuracy if a specific predictor was excluded. A greater decline in model accuracy would indicate that the excluded predictor is highly ranked in terms of importance [44]. In addition, the R-squared was used to identify the proportion of the variance in each outcome variable explained by the model predictors. Finally, partial correlation was used to determine the direction of the association between outcomes and predictors while taking control variables into account. The R package “caret” was used for data analysis [44].

## Results

A majority of our sample were female (*n* = 3676, 91%) with an average age of 40 years old (*SD* = 12 years) and 12 years of nursing experience (*SD* = 7 years). Over two-thirds of the sample worked in acute care sector (76%), compared to community (16%) and long-term care (7%) sectors, and in urban settings (68%), compared to suburban (18%) and rural (19%) areas.

Table 1 provides descriptive statistics as well as reliability indices for key predictors and outcomes. With the exception of life satisfaction, the mean for all of the key study variables is zero because factor scores from the CFA models used a centering approach. Table 2 presents the model fit indices for the six composite mental health outcomes. McDonald’ ω ranged between .74 to .93 indicating good internal consistency for 11 workplace factors and five mental health outcomes but acceptable internal consistency for two workplace factors (i.e., “job fit” and “growth development”) and one burnout indicator (i.e., personal accomplishment) (Table 1). The fit indices supported a good model fit for five of the mental health outcomes and an acceptable fit for one of these outcomes (i.e. PTSD) (Table 2).

**Table 2:** The model fit for the six composite mental health outcomes using CFA

Label	RSMEA	LL	UL	CFI	TLI	SRMR
Mental Health Outcomes						
Depression [9]	0.125	0.120	0.131	0.910	0.881	0.051
Anxiety [7]	0.122	0.155	0.129	0.960	0.940	0.033
PTSD [14]	0.120	0.117	0.123	0.861	0.835	0.055
Emotional Exhaustion [9]	0.149	0.144	0.155	0.905	0.873	0.060
Depersonalization [5]	0.154	0.142	0.166	0.931	0.862	0.059
Personal Accomplishment [8]	0.107	0.101	0.133	0.869	0.816	0.060

Table 3 demonstrates the results of the random forest regressions identifying the most important workplace conditions predicting nurse’s mental health. Overall, the model accounted for 18 to 37% of the variance across seven mental health outcomes. More specifically, *balance* (importance score: 28.80 to 43.28), *psychological protection* (importance score: 21.15 to 30.41) and *workload management* (importance score: 20.30 to 57.17) were the most important predictors of depression, anxiety, PTSD and emotional exhaustion. Furthermore, while *engagement* (importance score: 29.62), *workload management* (importance score: 26.21), *psychological protection* (importance score: 20.57) and *balance* (importance score: 20.45) were the most important predictors of depersonalization, *engagement (*importance score: 44.16*)* was the most important predictor of personal accomplishment. *Balance (*importance score: 38.11*)*, *psychological protection* (importance score: 25.91) and *engagement* (importance score: 24.99) were the most important predictors of life satisfaction. Additionally, the direction of associations between predictors and each outcome was examined. All predictors were negatively associated with negative mental health outcomes, and positively related to positive mental health outcomes (Fig. . 1). To be more specific, nurses’ reports of healthier workplace conditions predicted lower scores on depression, anxiety, PTSD and emotional exhaustion as well as higher scores on personal accomplishment and life satisfaction.

**Table 3:**
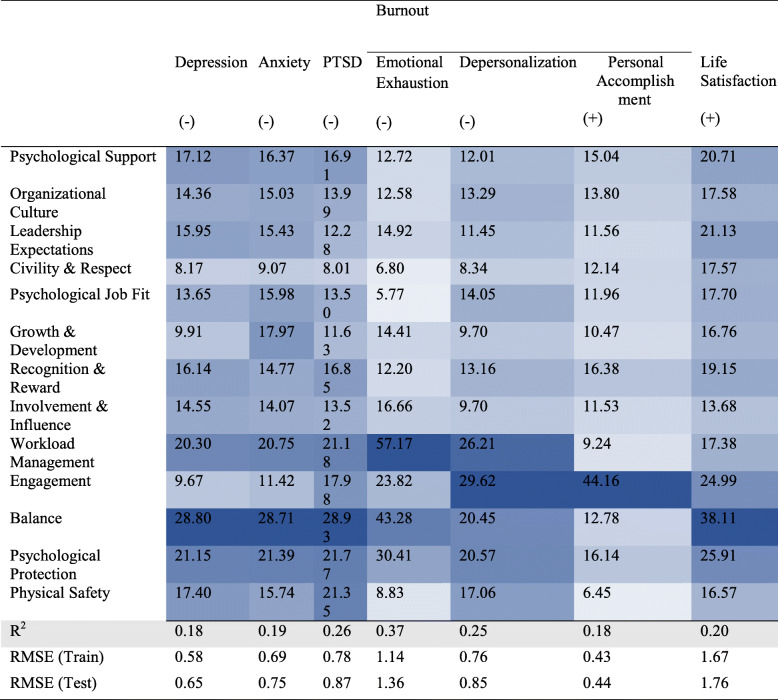
Workplace conditions regressed on mental health outcomes using random forest regression analyses

Note: The models are adjusted for demographics including age, gender, experience, healthcare sector and geographical region. The positive and negative signs refer to the direction of the bivariate association between predictors and outcomes

**Fig. 1:**
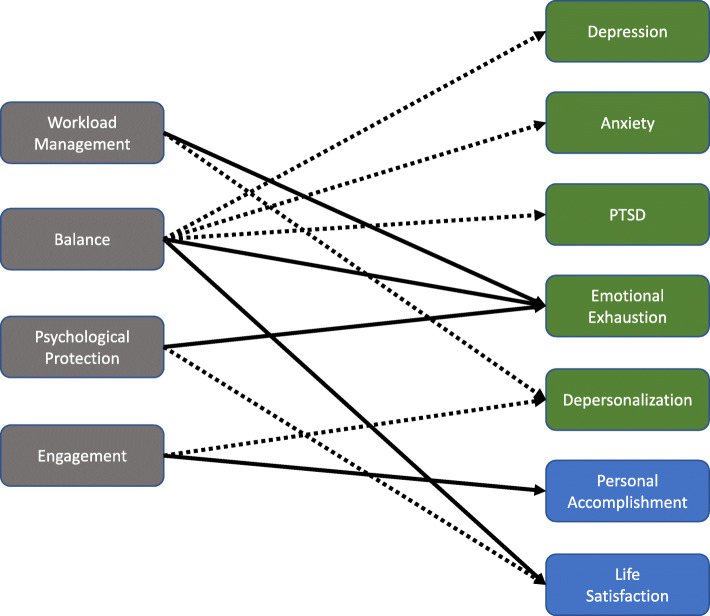
A diagram of key findings

## Discussion

To our knowledge, this is the first study to evaluate the work environment conditions predicting nurse mental health using the comprehensive and theoretically founded GMW. Our findings showed adequate worklife balance, psychological protection and workload management are the most important work environment conditions influencing nurse mental health. Worklife balance refers to work environments where employees have the flexibility and the autonomy to manage the demands of their professional and personal lives [31]. Worklife balance is not a component of the structural empowerment or magnet hospital bodies of evidence, but it is a component of the Area of Worklife Model’s control domain. When employees have greater control over work decisions, such as maintenance of worklife balance, they are less likely to report adverse mental health outcomes, including burnout [7, 29]. Of note is that previous research with younger generations of nurses identified that worklife balance is what they value most in their work environments [45].

Psychological protection represents work environments where employees’ psychological safety is ‘protected’ by preventing unnecessary stress, such as exposure to workplace violence and discrimination [31]. Although absent from the major models of nursing work environment, workplace violence literature has linked various types (e.g. physical, emotional) and sources (e.g. patients, co-workers) of workplace violence to poor nurse mental health [46, 47]. This finding is especially relevant with rising rates of workplace violence in healthcare [48].

Workload management reflects a work environment where assigned tasks and resources can be accomplished successfully within the time available [31]. This GMW factor is present within all three nursing work environment bodies of evidence where it is a known determinant of nurse and patient outcomes. In structural empowerment theory, workload management is represented by employees’ access to resources and supports in the workplace [22, 23]. The magnet hospital model conceptualizes the adequacy of staffing and resources as an indicator of workload management [6], and the Area of Worklife Model includes a workload dimension and describes it as working conditions where employee demands exceed their limits [7, 29].

In addition to these work environment conditions, engagement was an important predictor of nurse mental health outcomes, particularly personal accomplishment and life satisfaction. According to the GMW model, engagement reflects a work environment where employees have a sense of connection and commitment to their colleagues and the organization [31]. While this factor is conceptualized as an employee’s sense of community in the Areas of Worklife Model [29], it is not included in the structural empowerment and magnet hospital models of work environment. Consistent with our study, previous research has linked a greater sense of community and engagement to better nurse outcomes, including personal accomplishment [49].

In addition to enriching our theoretical understanding of healthy work environments, the study findings have profound implications for policy and practice, particularly in the context of a stressful and unprecedent pandemic that has overburdened healthcare workers around the globe with rising prevalence of mental health problems [17]. We found among all 13 GMW factors, worklife balance, psychological protection and workload management were the most important determinants of nurse mental health. These findings are especially important in light of a recent study describing BC nurses’ work environment using the GMW; this study found almost 75% of respondents were more concerned with these work environment factors than other workplace conditions [30].

These findings offer a direction for workplace strategies and interventions that best address current mental health needs of nurses. While worklife balance can be directly promoted through adopting certain strategies such as flexible scheduling systems that allow self- scheduling [50], we believe maintaining worklife balance is not possible without effective workload management. Previous research has taken a multidimensional approach to workload management where a variety of factors at the task level (e.g. interruptions when performing a task), work unit level (e.g. staffing levels, patient acuity and dependency) and job level (e.g. missed breaks, undone tasks) influence the magnitude of nurses’ workloads [51, 52]. A systems approach, therefore, is helpful when considering workload management factors that influence nurses’ mental health [51, 52].

Nurse staffing is one of the most well studied indicators of workload at the unit level [51]. A plethora of research evidence has linked inadequate staffing levels and inappropriate skill mix (i.e., types of healthcare providers) with work overload that results in negative nurse and patient outcomes [53]. While increasing the supply of professional nurses would be an important long-term goal, it is not an immediate intervention given the current shortage of nurses worldwide [53]. Nurse leaders must work with their current financial and human resources to enable better workload management. One of these interventions is evidence-based workload management tools that inform staffing guidelines based on patient needs in real time [53]. A systematic review has linked the use of such tools to better patient, organization and nurse outcomes including mental health [54].

Along with workload management, nurses are in need of work environments where their health and safety are protected. Workplace violence prevention strategies, including code white drills, enough security personnel, and employers that listen to staff’s suggestions are associated with nurses’ perceptions of increased workplace safety [55]. Given the rising prevalence of workplace violence in healthcare [48], policy makers and leaders must work towards instituting these evidence-based policies and interventions.

These recommendations are based on empirical data gathered from nurses using the GMW. The GMW survey corresponds with the Canadian “National Standard of Psychological Health and Safety”, the first international standard to comprehensively promote psychological health and safety in the workplace. This standard is accompanied with evidence-based resources and strategies to assist healthcare leaders with routine employee assessment and implementation of workplace interventions as needed [56].

### Strengths and limitations

This study used an innovative machine learning technique, random forest regression analysis, to identify the relative importance of 13 different workplace conditions in predicting nurse mental health. Compared to linear regression analysis, random forest regressions are more appropriate for analyzing and evaluating the relative importance of large numbers of predictors with a small sample size [57]. In such cases, random forest regression models yield more accurate results than conventional regression analyses [58]. Furthermore, the work environment measure used in this study, the GMW survey, is a comprehensive and validated instrument developed with the goal of improving workplace mental health [30]. Despite these strengths, the study also has some limitations including convenience sampling and low response rate which suggest the possibility of sampling bias. Additionally, the perspectives of non-practicing and non-unionized nurses, whose views and experiences may vastly differ from their actively working and unionized peers, were not included in this study. However, a descriptive comparison of our sample with the provincial nursing workforce demonstrated less than 10% difference with respect to nurse demographics including gender and professional designation (blinded for review). Despite this finding, the study results should be cautiously generalized to other samples (e.g. non-practicing, non-unionized nurses) and contexts (e.g. other jurisdictions). We also refrain readers from making any cause-and-effect conclusions due to the cross-sectional nature of the study.

## Conclusions

This is the first study examining the most important work environment predictors of nurse mental health using a validated and comprehensive measure of workplace conditions developed by the Mental Health Commission of Canada. Policy and practice efforts to promote nurse mental health should be data driven through routine and confidential assessment of work environment conditions and nurse mental health. This study suggested there is a critical need for addressing worklife balance, psychological protection and workload management in nurses’ work environments.

## Data Availability

The datasets analyzed during this study are not publicly available due to ethics approval guidelines but are available from the corresponding author on reasonable request and after obtaining the necessary ethics approvals.

## Abbreviations

AWS: Areas of Worklife Survey
BC: British Columbia
BCNU: British Columbia Nurses’ Union
CFA: Confirmatory Factor Analysis
CFI: Comparative Fit Index
DP: Depersonalization
EE: Emotional Exhaustion
GAD-7: General Anxiety Disorder-7
GMW: Guarding Mind at Work
CWEQ-II: Conditions of Work Environment Questionnaire II
MBI-HSS: Maslach Burnout Inventory-Human Services Survey
PA: Personal Accomplishment
PHQ-9: Patient Health Questionanire-9
PTSD: Post-Traumatic Stress Disorder
PTSS-14: Post-Traumatic Stress Syndrome 14-Questions Inventory
PES-NWI: Practice Environment Scale from the Nursing Work Index
RF: Random Forest
RMSE: Root Mean Square Error
SRMR: Standardized Root Mean Squared Residual
TLI: Tucker Lewis Index
